# A Method to Minimise the Impact of ECG Marker Inaccuracies on the Spatial QRS-T angle: Evaluation on 1,512 Manually Annotated ECGs

**DOI:** 10.1016/j.bspc.2020.102305

**Published:** 2021-02

**Authors:** William J. Young, Stefan van Duijvenboden, Julia Ramírez, Aled Jones, Andrew Tinker, Patricia B. Munroe, Pier D. Lambiase, Michele Orini

**Affiliations:** a Clinical Pharmacology Department, William Harvey Research Institute, Barts and the London School of Medicine and Dentistry, Queen Mary University of London, EC1M 6BQ, United Kingdom; b Institute of Cardiovascular Sciences, University of College London, WC1E 6BT, United Kingdom; c Barts Heart Centre, St Bartholomew’s Hospital, Barts Health NHS trust, London, EC1A 7BE, United Kingdom

**Keywords:** Electrocardiogram, Vectorcardiogram, Spatial QRS-T angle, Automatic analysis, Population distribution

## Abstract

The spatial QRS-T angle (QRS-Ta) derived from the vectorcardiogram (VCG) is a strong risk predictor for ventricular arrhythmia and sudden cardiac death with potential use for mass screening. Accurate QRS-Ta estimation in the presence of ECG delineation errors is crucial for its deployment as a prognostic test. Our study assessed the effect of inaccurate QRS and T-wave marker placement on QRS-Ta estimation and proposes a robust method for its calculation.

Reference QRS-Ta measurements were derived from 1,512 VCGs manually annotated by three expert reviewers. We systematically changed onset and offset timings of QRS and T-wave markers to simulate inaccurate placement. The QRS-Ta was recalculated using a standard approach and our proposed algorithm, which limits the impact of VCG marker inaccuracies by defining the vector origin as an interval preceding QRS-onset and redefines the beginning and end of QRS and T-wave loops.

Using the standard approach, mean absolute errors (MAE) in peak QRS-Ta were >40% and sensitivity and precision in the detection of abnormality (>105°) were <80% and <65% respectively, when QRS-onset was delayed or QRS-offset anticipated >15 ms. Using our proposed algorithm, MAE for peak QRS-Ta were reduced to <4% and sensitivity and precision of abnormality were >94% for inaccuracies up to ±15 ms. Similar results were obtained for mean QRS-Ta.

In conclusion, inaccuracies of QRS and T-wave markers can significantly influence the QRS-Ta. Our proposed algorithm provides robust QRS-Ta measurements in the presence of inaccurate VCG annotation, enabling its use in large datasets.

## Highlights

• Inaccuracies of QRS and T-wave markers significantly impact QRS-Ta estimation.
• These errors influence the classification of clinically relevant abnormal values.
• Our algorithm provides robust measurements in the presence of inaccurate VCG markers.
• We present for the first time, the distribution of the QRS-Ta in a large cohort.

## 1 Introduction

With the availability of increasingly large data-sets and improved computational abilities, there has been renewed interest in the utility of markers derived from the vectorcardiogram (VCG) such as the spatial QRS-T angle (QRS-Ta), to improve cardiovascular risk prediction [1]. A widened QRS-Ta is associated with an increased risk of ventricular arrhythmia, sudden cardiac death and cardiac-related mortality in general and clinical populations [[2], [3], [4], [5]]. The QRS-Ta has potential to improve clinical decision making in patients with ischaemic heart disease, as abnormal values are associated with occurrence of life-threatening ventricular arrhythmias, thus identifying those who may benefit most from an implantable cardioverter-defibrillator (ICD) [6]. Additionally, the growth of biobanks and collaborations through consortia have enabled new opportunities for the epidemiological and clinical evaluation of ECG markers, such as genetic analyses improving our understanding of the underlying biology [7]. To capitalise on these rich datasets and facilitate the application of the QRS-Ta in routine clinical practice, the ability to reliably analyse large volumes of electrocardiograms (ECGs) with minimal human intervention is crucial as manual annotation of tens of thousands of ECGs is impractical.

The VCG can be derived from the 12-lead ECG using standard transformations [8]. The QRS-Ta mean and peak are subsequently calculated as the angle between the spatial QRS and T-wave loop mean and maximal amplitudes, respectively. To date, clear details of algorithms used to derive the QRS-Ta have not been widely shared and key aspects of the algorithm remain unscrutinised. Recently, efforts have been made to develop a harmonised approach to improve the consistency of measurements for some aspects of its calculation [9]. However, a crucial aspect yet to be formally assessed is the impact of errors in QRS and T-wave marker placement on the QRS-Ta (which in standard algorithms determines the origin, start and end of the spatial loops). This needs to be evaluated for the development of algorithms designed to fully automatically analyse large datasets.

The aims of this study were to 1) Evaluate the impact of QRS and T-wave marker placement on the accuracy of peak and mean QRS-Ta; 2) Propose and test a simple but robust algorithm to provide fully-automated measurements of the QRS-Ta in the presence of inaccurate QRS and T-wave marker placement, comparing performance against manual annotation by 3 expert reviewers and the current standard algorithm; 3) Make this algorithm publicly available for use; 4) Provide the distribution of the QRS-Ta in a population of over 34,000 unselected individuals.

## 2 Methods

### 2.1 Study population

The UK Biobank (UKB) is a prospective study of 488,377 volunteers aged 40-69 years at recruitment (2006-2008). The UKB study has approval from the North West Multi-Centre Research Ethics Committee, and all participants provided informed consent [10]. The number of ECGs for which a signal-averaged VCG could be obtained after discarding corrupt or incomplete files was 35,501. These were 10-second 12-lead ECGs from the imaging sub-cohort, recorded at a follow up visit (May 2014–September 2019). The project was supported under application 8256 to UK Biobank.

### 2.2 Initial ECG Data Analysis

All analyses were carried out using Matlab 2018b. The sampling rate of ECGs was 500 Hz. A bandpass Butterworth filter was applied ([0.5, 45]) for noise reduction, while limiting the impact on QRS morphology. Signal averaging was implemented to further reduce noise as in previous studies [11]. Signal-averaged ECGs were produced using only beats with similar morphology, identified as those for which the first principal component showed a correlation coefficient >0.80 with respect to the median beat. ECGs were excluded if fewer than five cardiac beats were available for generating the averaged beat. Orthogonal X, Y and Z leads were estimated from the 12-lead signal-averaged beat using Kors’ regression matrix [12]. The VCG was derived as the representation of the cardiac cycle as a trajectory of the X, Y and Z leads over time:

(1) $$\vec{v}(t)=\left(x\left(t\right),\;y\left(t\right),\;z(t)\right)$$

Baseline correction was subsequently applied to limit the impact of baseline wander on marker placement and construction of QRS and T wave loops.

### 2.3 Calculation of “reference” QRS-Ta values

A total of 1,512 ECGs were chosen at random and two cardiologists independently manually annotated the VCG markers. One QRS onset (QRSon), QRS end (QRSend) and T-wave end (Tend) marker was manually annotated per VCG (i.e. multi-lead annotation), using an ad-hoc Graphical User Interface in Matlab customized from previous studies [13,14]. A third observer reviewed the VCGs if the absolute difference between the markers was >15 ms. Reference VCG markers were defined as the average between the manually annotated markers by the first two reviewers if their absolute difference was ≤15 ms and as the manually annotated markers by the third reviewer if their absolute difference was >15 ms. Reference values for the spatial QRS-Ta were obtained following the standard approach using these annotated markers as described next.

### 2.4 Standard approach for QRS-Ta estimation

The origin of both the QRS and T-wave loops for the identification of the QRS and T-wave vectors is taken at ${\vec{v}}_{0}=\vec{v}(t_{0})$, where $t_{0}={QRS}_{ON}$. The QRS and T-wave loops are then defined by vectors:

(2) $${\vec{v}}_{QRS}\left(t\right)=\vec{v}\left(t\right)-{\vec{v}}_{0},\;\text{w}\text{i}\text{t}\text{h}\;\;{QRS}_{ON}\leq t\leq {QRS}_{END},$$

(3) $${\vec{v}}_{TW}\left(t\right)=\vec{v}\left(t\right)-{\vec{v}}_{0}\;,\text{w}\text{i}\text{t}\text{h}\;{QRS}_{END}<t\leq T_{END.}$$

The peak vectors ${\vec{v}}_{QRS}^{max}$ and ${\vec{v}}_{TW}^{max}$ are defined as those associated with the largest magnitude, i.e. ${\vec{v}}_{QRS}^{max}={\vec{v}}_{QRS}\left(t_{m}\right)$, with $t_{m}=\text{a}\text{r}\text{g}(\text{m}\text{a}\text{x}(\left\|{\vec{v}}_{QRS}(t)\right\|))$, and ${\vec{v}}_{TW}^{max}={\vec{v}}_{TW}\left(t_{m}\right)$, with $t_{m}=\text{a}\text{r}\text{g}(\text{m}\text{a}\text{x}(\left\|{\vec{v}}_{TW}(t)\right\|))$. The mean vectors ${\vec{v}}_{QRS}^{mean}$ and ${\vec{v}}_{TW}^{mean}$ are measured as the temporal mean of ${\vec{v}}_{QRS}\left(t\right)$ and ${\vec{v}}_{TW}\left(t\right)$. The spatial peak QRS-Ta, ($\alpha_{P}$), is measured as the angle between ${\vec{v}}_{QRS}^{max}$ and ${\vec{v}}_{TW}^{max}$ while the spatial mean QRS-Ta, ($\alpha_{M}$), is measured as the angle between ${\vec{v}}_{QRS}^{mean}$ and ${\vec{v}}_{TW}^{mean}$.

### 2.5 Proposed robust approach for QRS-Ta estimation

**Fig. 1:**
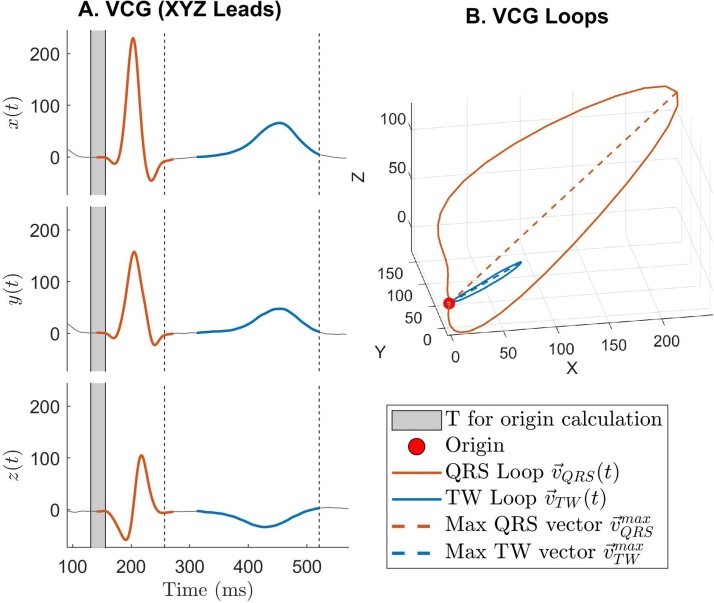
QRS-T angle measurement using our proposed approach. A: X, Y and Z leads showing the QRS and T-wave in orange and blue, respectively. The solid grey block prior QRS complex represents the interval for the calculation of the vectors’ origin. B: VCG loops in the XYZ space. The red dot represents the vectors’ origin and the dashed lines the peak QRS and T-wave vectors.

The standard approach described above may be inaccurate when there are errors in the measurement of QRS and T-wave onset and offset. In our proposed approach we implement improvements, based on the results of sensitivity analyses carried out to assess the impact of VCG marker inaccuracies on the QRS-Ta in this study (as described in results section 3.1). Critical sources of QRS-Ta estimation errror include: inaccuracy in the positioning of the loops origin, delay in the onset of the QRS loop, anticipation in the onset of T-wave loop and impact of noise on low-amplitude T-waves. Our proposed algorithm (Fig. 1) implements the following amendments to minimize the impact of these issues:

1. The origin of both loops is taken as the median value of $\vec{v}\left(t\right)$ over a short interval preceding QRSon:
  (4) $${\vec{v}}_{0}=median\left(\vec{v}\left(t\right)\right),\;\text{w}\text{i}\text{t}\text{h}\;\left({QRS}_{ON}-\tau_{0}\right)\leq t\leq {QRS}_{ON}$$
  This is to identify in a robust manner an interval when there is limited electrophysiological activity, which does not significantly influence the amplitude of the QRS loop and subsequently the estimation of the QRS-Ta. In this study, $\tau_{0}$ = 25 ms. This short window (QRS_ON_ – $\tau_{0}$ : QRS_ON_) was chosen to avoid encroachment into either the P-wave or the QRS complex, with the latter being particularly problematic and a vulnerability when using the standard approach. If either the end of the P wave or the beginning of the QRS complex are within this window, taking the median amplitude as the time point for the vector origin will reduce the risk of these regions influencing its location.
2. The onset and end of the QRS loop are modified such that:
  (5) $${\vec{v}}_{QRS}\left(t\right)=\vec{v}\left(t\right)-{\vec{v}}_{0},\;\text{w}\text{i}\text{t}\text{h}\;\left({QRS}_{ON}-\tau_{QRS}^{ON}\right)\leq t\leq \left({QRS}_{END}+\tau_{QRS}^{END}\right)$$
  These time points before QRS onset and after QRS end contribute very little to the formation of the loop and the calculation of its peak and mean amplitudes, and therefore can be used as a ‘buffer’ in case of inaccurate marker placement. A value of $\tau_{QRS}^{ON}=\tau_{QRS}^{END}=$ 15 ms was chosen based on the results from this study investigating the impact of marker placement on QRS-Ta calculation, that show that QRS-Ta estimates can be severely affected when error in marker placement results in longer QRSon and shorter QRSend (see results section 3.2 and Fig. 2).

**Fig. 2:**
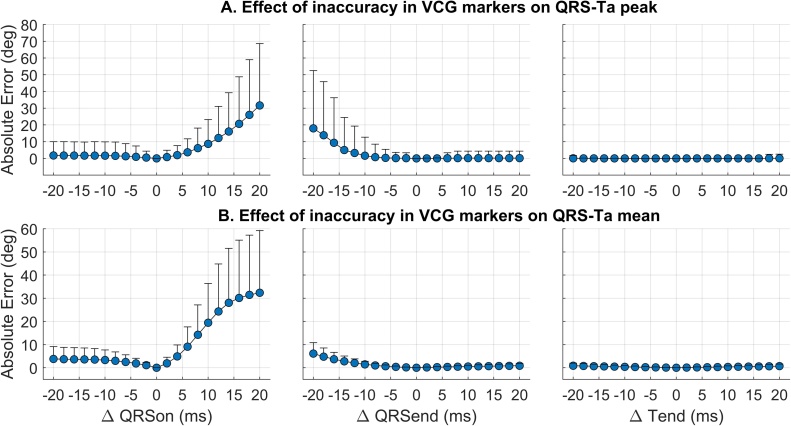
Effect of systematically moving manually annotated reference VCG markers within ± 20 ms, on the estimation error of peak (A) and mean (B) QRS-Ta using the standard approach. Markers and bars represent the mean and standard deviation of the absolute error. From left to right, changes were made to QRSon, QRSend or Tend only.
3. The beginning of the T wave loop is delayed:
  (6) $${\vec{v}}_{TW}\left(t\right)=\vec{v}\left(t\right)-{\vec{v}}_{0},\;\text{w}\text{i}\text{t}\text{h}\;\left({QRS}_{END}+\tau_{TW}^{ON}\right)\leq t\leq T_{END}$$
  This is to ensure no part of the QRS loop is included in the construction of the T wave loop should, for example, QRS end be delayed by inaccurate marker placement. The value $\tau_{TW}^{ON}=$ 40 ms was chosen following review of a random sample of VCG signals and their constructed QRS and T-wave loops, for example as shown in Fig. 1. The core information forming the T wave loop is centred around the T-wave peak (see results section 3.1), which is retained using this window.
4. No attempt to measure QRS-Ta is made if:
  (7) $$R=\left\|{\vec{v}}_{TW}^{max}\right\|/\left\|{\vec{v}}_{QRS}^{max}\right\|<0.10$$
  In our analysis, we observed that T waves showing extreme low amplitudes require manual revision as automated marker placement is significantly more likely to be inaccurate. Although this condition is rarely verified, it has an impact on accurate identification of abnormal QRS-Ta.

### 2.6 Simulation study to assess the effect of VCG marker placement

To evaluate the impact of inaccuracies of marker placement on the spatial QRS-Ta, QRSon, QRSoff and Tend markers were systematically moved in 2 ms steps from −20 ms to +20 ms from the manually annotated reference and the QRS-Ta was recalculated using the standard approach. The mean absolute error (MAE) with respect to the reference QRS-Tas was assessed. This was repeated, using our proposed robust algorithm to calculate the QRS-Ta using the annotated VCG markers, and the results compared. Furthermore, sensitivity and precision in the detection of clinically relevant abnormality in the QRS-Tas, i.e. $\alpha_{P}>105{}^{\circ}$ or $\alpha_{M}>105{}^{\circ}$, were assessed using both algorithms [1].

The effect of noise on the 10-seconds ECG recordings was assessed in simulation studies. White Gaussian noise was added to each lead of the raw ECGs and the full algorithm, including signal averaging, VCG derivation, automatic VCG markers delineation and spatial QRS-Ta calculation, was re-implemented for all 1,512 ECGs. The entire process was repeated 5 times per recording. At each iteration, the amplitude of the noise added to each ECG lead was increased in such a way to obtain a signal-to-noise ratio (SNR) decreasing from 20 dB to 0 dB in steps of 5 dB.

### 2.7 Real data analysis on a large dataset

We subsequently calculated the QRS-Ta on our full dataset (N = 35,501) and plotted its distribution. To facilitate implementation of our proposed robust algorithm in this large dataset we use automatic identification of QRS and T-wave fiducial points, using a simple algorithm combining specific criteria on the magnitude of the XYZ leads and its derivative. Our proposed robust algorithm for QRS-Ta calculation along with example ECG files, is provided with this manuscript in the accompanying supplementary materials. For reproducibility, we also include a description of the automatic QRS and T-wave marker algorithm with these files.

## 3 Results

### 3.1 Assessment of marker placement on spatial QRS-Ta

Manually annotated VCG markers from the first two expert reviewers showed high correlation (*ρ > 0.926*) and small MAEs. Reannotation by a 3^rd^ Reviewer was required in 13 VCGs (0.9%) for QRSon, 35 (2.3%) for QRSend and 164 (10.8%) for Tend. Using these reference markers, values for the QRS-Ta in the reference population (N = 1,512) were 28.8°, 17° / 44.6° (median, 1^st^ / 3^rd^ quartiles) for $\alpha_{P}$ and 41.1° (25.2° / 63.9°) for $\alpha_{M}$.

**Fig. 3:**
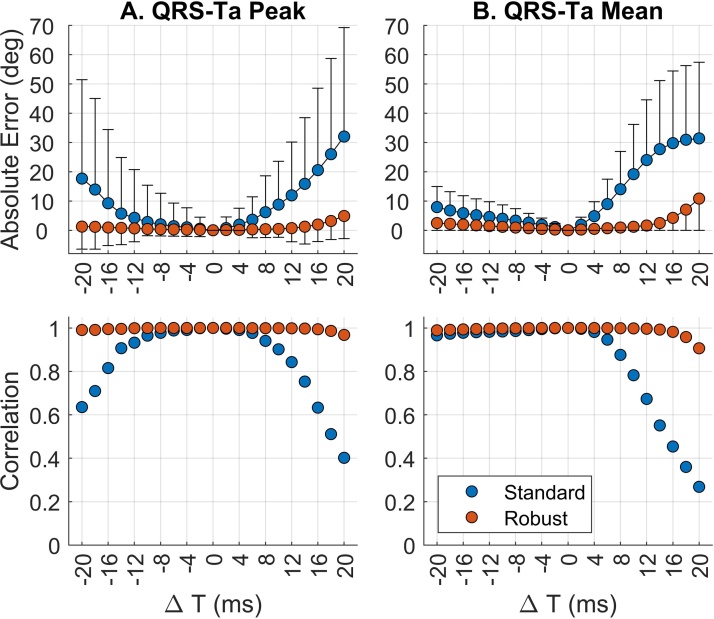
Effect of moving all three reference VCG markers, on the estimation error of peak (A) and mean (B) QRS-T angles using standard (blue) and our proposed robust (red) approaches. Top two sub-plots: markers and bars represent the mean and standard deviation of the absolute error. Bottom two sub-plots: change in correlation coefficient between reference QRS-Ta (time point 0) and QRS-Ta calculated having systematically shifted the VCG marker. In this simulation, QRSon, QRSend and Tend were moved simultaneously in the same direction.

In this simulation study, inaccuracies in VCG annotations have a strong impact on the QRS-Ta measured with the standard approach. Fig. 2 shows variation of QRSon, QRSend and Tend within ± 20 ms, results in mean absolute errors up to 32° (158% in terms of relative error) for $\alpha_{P}$ and 32° (103%) for $\alpha_{M}$. The strongest effect on estimation error was delay of QRSon and anticipation of QRSend, while Tend inaccuracies had only a small effect (Fig. 2). This was accompanied with a marked reduction in the correlation coefficient with the reference QRS-Ta value (T = 0 ms), as VCG markers were progressively moved from their annotated position (Fig. 3, blue markers). Inspection of Fig. 2 shows anticipation of QRS onset or delay of QRS offset, has no significant influence on the resulting QRS-T angle calculated. In comparison, using our proposed robust algorithm, the sensitivity of QRS-Ta estimation to inaccuracies in VCG annotations was dramatically reduced and the correlation coefficient remains high throughout (Fig. 3, red markers).

**Fig. 4:**
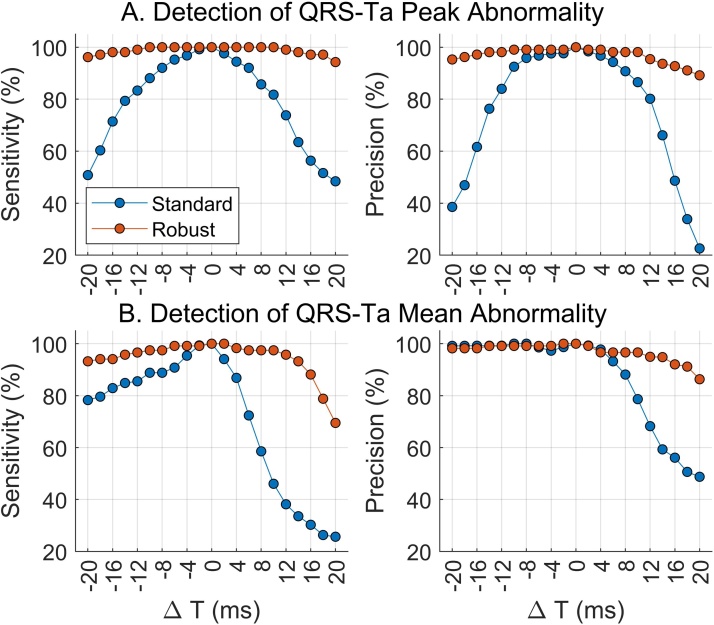
QRS-T angles for all 1,512 ECGs were measured using standard (blue) and robust(red) algorithms after systematically moving the manually annotated reference VCG markers in 2 ms steps until ± 20 ms. Sensitivity and precision of abnormal angle detection ($\alpha_{P}$ or $\alpha_{M}$ >105 deg) were assessed using standard (blue) and robust (red) algorithms after moving reference VCG markers in 2 ms steps. In this simulation, QRSon, QRSend and Tend were moved simultaneously in the same direction.

**Fig. 5:**
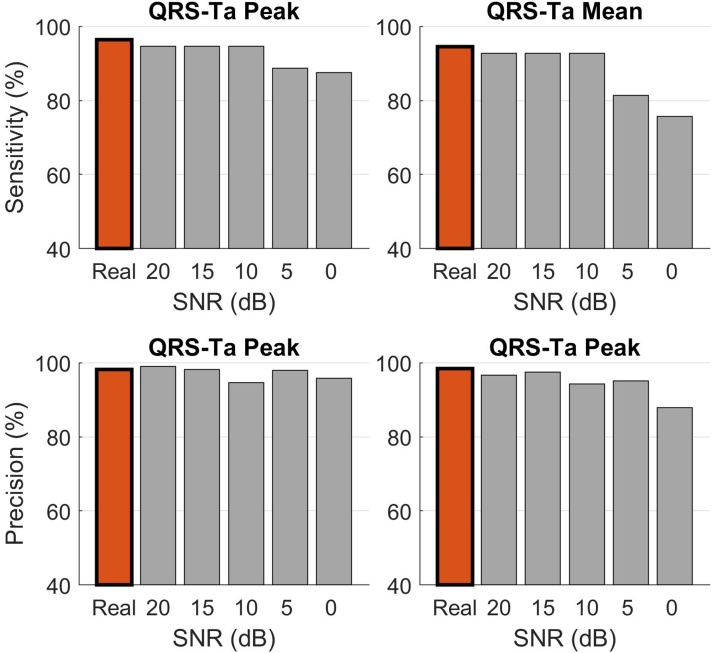
Effect of noise on the detection of abnormal QRS-T angle using the robust approach. QRS-T angles for all 1,512 ECGs were measured after white noise was added to the 10 sec ECG recordings and sensitivity and precision of abnormal angle detection ($\alpha_{P}$ or $\alpha_{M}$ >105 deg) were assessed for both peak (left) and mean (right) QRS-T angles.

Using the standard approach, inaccuracies in VCG annotations significantly impaired the ability to detect abnormal QRS-Tas, which could have important consequences in its use for clinical application. For some configurations, sensitivity and precision significantly dropped below 50% (Fig. 4). Our proposed robust approach however, ensured detection of an abnormal QRS-Ta with high sensitivity and precision. Comparing our reference QRS-Ta values calculated using standard and robust algorithms, a high correlation was observed (*p* > 0.987 and *p* > 0.988 for $\alpha_{P}$ and $\alpha_{M}$ respectively). The correlation coefficients between automated and reference values remained >0.94 for SNR ≥ 5 dB. Detection of abnormal angles was also only marginally affected by noise, with sensitivity, specificity and precision above 94.2% for both $\alpha_{P}$ and $\alpha_{M}$ for SNR ≥ 10 dB (Fig. 5).

Out of 1,512 ECGs with manual annotation, 56 (3.7%) showed $R=\left\|{\vec{v}}_{TW}^{max}\right\|/\left\|{\vec{v}}_{QRS}^{max}\right\|<0.10$, indicating a flat T-wave. To assess the impact of low T-wave amplitude on the accuracy of QRS-Ta estimation, we defined outliers as those QRS-Ta estimates with absolute error larger than the median absolute error plus 3 times the inter-quartile range ($\left|e\right|>\text{m}\text{e}\text{d}\text{i}\text{a}\text{n}\left|e\right|+3\times \text{I}\text{Q}\text{R}\left|e\right|$). We found that VCGs showing R < 0.10 were significantly more likely to produce outliers than the rest, with odds ratio equal to 12.4 (9.92–22.4), P = 9.6 × 10^−14^, for $\alpha_{P}$ and 9.6 (5.1–18.0), P = 6.9 × 10^−10^ for $\alpha_{M}$ (Fisher's exact test).

### 3.2 Real-data analysis in a large cohort

**Fig. 6:**
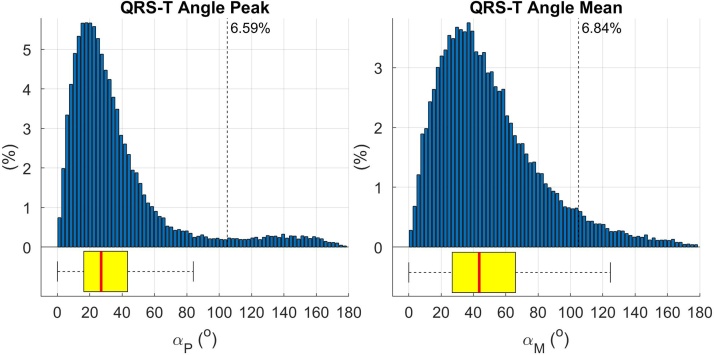
Histogram showing peak and mean QRS-T angle distributions for 34,150 individuals with a 12-lead ECG recorded in the UK Biobank study. Vertical dotted line shows cut off for abnormality (> 105°).

A histogram showing the distribution of the spatial QRS-Ta for 34,150 individuals is shown in Fig. 6. The median age at the time of ECG acquisition was 64 years (58–82) and 51.7% were female. The median (1^st^–3^rd^ quartiles) mean and peak QRS-Ta were 43.07°, (26.6°–65.1°) and 26.7°, (16.1°–42.6°), respectively, with 6.36% and 6.23% abnormality.

## 4 Discussion

The main results of this study are: (1) The QRS-Ta can be significantly affected by inaccuracies in annotation of VCG markers when using a standard algorithm, especially QRS onset and end impacting the identification of clinically relevant abnormal QRS-Ta values. (2) Our proposed algorithm is robust and abnormal QRS-Ta values are identified with significant improvements in sensitivity and precision in the setting of inaccurate VCG marker placement. (3) The prevalence of clinically abnormal mean and peak QRS-Ta in a sample of over 34,000 unselected individuals from UK Biobank is 6.36% and 6.23% respectively.

The QRS-Ta is an important clinical risk marker for ventricular arrhythmia and sudden cardiac death and the detection of abnormal values has potential to significantly improve risk stratification of patients and guide medical and interventional strategies to prevent morbidity and reduce mortality [[2], [3], [4], [5], [6]]. Additionally, new opportunities are available to take advantage of the ever growing number of biobanks and consortia, to improve our understanding of the biological mechanisms involved, as reported previously and for other ECG derived parameters [7,15,16].

Determining the exact location of QRS and T wave fiducial points represents a significant challenge in itself and is prone to error. This is the first study to explore the impact of VCG markers placement on the QRS-Ta and important aspect to consider to facilitate its clinical application and investigation in large epidemiological studies. We have used a large manually annotated dataset by 3 reviewers to run this simulation study. We show that, when using a standard algorithm, inaccuracies of markers can significantly influence the resulting QRS-Ta, with incorrect placement of QRS onset or offset producing the larger estimation errors. A small anticipation of QRSend or delay in QRSon will significantly modify the QRS loop resulting in the loss of important ECG signal information within the QRS complex, leading to inaccuracy in the estimation of the QRS loop peak and mean amplitudes. Additionally, if the standard approach of identifying the vectors’ origin with QRSon is used, delay of QRS onset will result in a change in the position of the vectors’ origin, which in turn significantly impacts the resulting QRS-Ta calculated. While inaccuracies in the annotation of Tend has been shown to be less problematic, VCG exhibiting very low amplitude or flat T-waves (i.e. with $R=\left\|{\vec{v}}_{TW}^{max}\right\|/\left\|{\vec{v}}_{QRS}^{max}\right\|<0.10$) were associated with a significantly higher propensity to large errors and should therefore be highlighted for manual inspection. This only affected 3.3% of our total cohort.

Our analyses show that redefinition of the vectors’ origin to a region with minimal electrophysiological activity, along with QRS and T-wave loop onset and end modification to introduce a window ‘buffer’, makes the estimation of QRS-Ta far less sensitive to VCG marker inaccuracies. This is a key feature for automated analysis of large data sets. The intervals used to define the vector origin and beginning/end of QRS and T wave loops in our algorithm have been chosen based on the results of this study. Our data shows that intervals outside of the QRS complex and the peak of the T-wave, contribute little to QRS-Ta estimation, and thus including a buffer outside of these regions, still results in QRS-Ta measurements which are highly correlated with reference values. Our proposed algorithm may also be beneficial for beat to beat analyses without signal averaging, where noise may influence fiducial point placement.

Definition of the vector origin point has previously been considered with respect to its impact with clinical outcomes. Perez-Alday et al identified differences in spatial QRS-Ta measurements and improvement in its predictive accuracy as a biomarker for sudden cardiac death, when comparing the isoelectric point (either T-P interval or P-R interval if T-P interval is not suitable) as the vector origin compared with QRS onset (considered to be the industry standard) [9]. In this study, we have demonstrated that incorrect marker placement when using QRS onset as the vector origin, is likely to result in significant inaccuracies in the measurement of the spatial QRS-Ta which may explain the findings in their study. We chose to calculate the loops origin within a short interval preceding the QRS onset as we observed that significant baseline wander and / or noise can be present in T-P interval. Additionally, ECG software used by biobanks may only output averaged cardiac beats for each lead, thus limiting the ability to use T-P interval.

While mean ± SD or median (IQR) of QRS-Ta has been published in the literature by some cohorts, a histogram distribution as provided in this paper, has not previously been reported. The histogram (Fig. 6) shows significant skew to the right. The ranges reported in our cohort are comparable to those in other general population studies [1]. To facilitate additional study and a harmonisation to the approach of calculating the spatial QRS-T angle, we have made our algorithms freely available for use along with example ECG data (see Supplementary materials).

This study has some limitations. The proposed algorithm ensures greater robustness against the effect of imprecision in the VCG markers placement. However, this is achieved at the expense of introducing a very small bias in the case the marker placement is precise (Fig. 3). Specific but rare situations may require modification of values chosen for defining the onset and offset of VCG loops in our proposed robust algorithm. These include a paced rhythm, significant ST segment deviation and the presence of a delta wave with an accompanying short P-R interval. However, as $\tau_{0}$, $\tau_{QRS}^{ON}$, $\tau_{QRS}^{END}$ and $\tau_{TW}^{ON}$ have been chosen to fall well within the normal ranges and have been tested in this large study, we anticipate that they are generalizable to the adult population. The UK Biobank cohort with 12-lead ECGs does not have a follow up period to evaluate the impact of our algorithm on cardiovascular outcomes; however, we have demonstrated a good precision for the classification of individuals to an abnormal or normal spatial QRS-Ta. Further testing of this algorithm for the evaluation of clinical outcomes is warranted. Further studies should also focus on the interaction of the spatial QRS-Ta with other markers of spatio-temporal heterogeneity of repolarization in large cohorts and compare their prognostic value [[17], [18], [19], [20], [21]].

## 5 Conclusion

Inaccuracies of QRS and T-wave markers can significantly influence the QRS-Ta and impact the detection of abnormal physiological values, limiting its clinical application and investigation in large scale epidemiological studies. Our proposed algorithm provides robust QRS-Ta measurements in the presence of inaccurate VCG annotation by redefinition of the vectors’ origin and loops onset/end and can significantly reduce the amount of human intervention required to obtain reliable results. In addition, we present for the first time, the distribution of the QRS-Ta in a large cohort and make the algorithm available for application in other datasets.

## CRediT authorship contribution statement

**William J. Young:** Conceptualization, Data curation, Formal analysis, Writing - review & editing. **Stefan van Duijvenboden:** Methodology, Writing - review & editing. **Julia Ramírez:** Methodology, Writing - review & editing. **Aled Jones:** Data curation, Writing - review & editing. **Andrew Tinker:** Supervision, Funding acquisition, Writing - review & editing. **Patricia B. Munroe:** Supervision, Funding acquisition, Writing - review & editing. **Pier D. Lambiase:** Supervision, Funding acquisition, Writing - review & editing. **Michele Orini:** Conceptualization, Methodology, Data curation, Formal analysis, Supervision, Funding acquisition, Writing - review & editing.
